# Genome-wide tiled detection of circulating Mycobacterium *tuberculosis* cell-free DNA using Cas13

**DOI:** 10.1038/s41467-023-37183-8

**Published:** 2023-03-31

**Authors:** Sri Gowtham Thakku, Jackson Lirette, Kanagavel Murugesan, Julie Chen, Grant Theron, Niaz Banaei, Paul C. Blainey, James Gomez, Sharon Y. Wong, Deborah T. Hung

**Affiliations:** 1 Broad Institute of MIT and Harvard, Cambridge, MA USA; 2 Department of Pathology, Stanford University School of Medicine, Stanford, CA USA; 3 DSI-NRF Centre of Excellence for Biomedical Tuberculosis Research and SAMRC Centre for Tuberculosis Research, Division of Molecular Biology and Human Genetics, Faculty of Medicine and Health Sciences, Stellenbosch University, Cape Town, South Africa; 4 Department of Medicine, Division of Infectious Diseases and Geographic Medicine, Stanford University School of Medicine, Stanford, CA USA; 5 Clinical Microbiology Laboratory, Stanford Health Care, Palo Alto, CA USA; 6 Department of Biological Engineering, Massachusetts Institute of Technology, Cambridge, MA USA; 7 Koch Institute for Integrative Cancer Research at MIT, Cambridge, MA USA; 8 Department of Genetics, Harvard Medical School, Boston, MA USA; 9 Department of Molecular Biology and Center for Computational and Integrative Biology, Massachusetts General Hospital, Boston, MA USA

**Keywords:** Infectious-disease diagnostics, Pathogens, Assay systems

## Abstract

Detection of microbial cell-free DNA (cfDNA) circulating in the bloodstream has emerged as a promising new approach for diagnosing infection. Microbial diagnostics based on cfDNA require assays that can detect rare and highly fragmented pathogen nucleic acids. We now report WATSON (Whole-genome Assay using Tiled Surveillance Of Nucleic acids), a method to detect low amounts of pathogen cfDNA that couples pooled amplification of genomic targets tiled across the genome with pooled CRISPR/Cas13-based detection of these targets. We demonstrate that this strategy of tiling improves cfDNA detection compared to amplification and detection of a single targeted locus. WATSON can detect cfDNA from Mycobacterium *tuberculosis* in plasma of patients with active pulmonary tuberculosis, a disease that urgently needs accurate, minimally-invasive, field-deployable diagnostics. We thus demonstrate the potential for translating WATSON to a lateral flow platform. WATSON demonstrates the ability to capitalize on the strengths of targeting microbial cfDNA to address the need for point-of-care diagnostic tests for infectious diseases.

## Abstract (web summary)

In this work, the authors developed a multiplexed, minimally invasive, CRISPR-Cas13-based approach to detect *Mycobacterium tuberculosis* cell-free DNA in the plasma of active pulmonary tuberculosis patients.

## Introduction

Infectious diseases remain a tremendous burden on the global health system. In 2019, an estimated 7.7 million people died from an infection, accounting for 14% of all deaths globally that year^1^. Respiratory and diarrheal infections, along with tuberculosis, HIV/AIDS and malaria, continue to take their toll amidst the constant threat of emerging pathogens, such as SARS-CoV-2, which has claimed over 5 million lives since its inception^2^. In response to the COVID-19 pandemic, highly sensitive diagnostic tests were rapidly developed by targeting the SARS-CoV-2 genome, leveraging the programmability of nucleic acid detection. However, unlike SARS-CoV-2, which can be reliably detected using a non-invasive nasopharyngeal swab, many infections can require more challenging, even potentially onerous and invasive sample collection, and yet still may elude diagnosis altogether.

The need for sensitive, specific, and simple diagnostics for infectious diseases has never been clearer. Recently, biological and technical advances have enabled the use of liquid biopsies, wherein biomarkers of disease are detected in readily-accessible bodily fluids, like blood and urine, to obtain critical diagnostic information for a range of human conditions. Cell-free human DNA in plasma is proving to be an invaluable biomarker in prenatal screening, oncology, toxicology and transplant medicine, where its detection and characterization in the peripheral circulation provides critical information on processes occurring in deeper, harder to access tissues. Along these lines, circulating microbial DNA may also be an easily accessible biomarker that enables the diagnosis of infections deep within the body, bypassing the need for biopsies or other difficult or invasive sample collection techniques. This modality of sample collection and pathogen biomarker detection – ideally performed at the point-of-care – could transform infectious disease management. Indeed, blood-based nucleic acid tests (NATs) are well established for detecting viral infections such as HIV^3^ and hepatitis C^4^. Historically however, such tests have played little role in the diagnosis of non-viral infections such as bacteria, in large part because of the relative scarcity of pathogen nucleic acids in blood for other infection types. Recently, polymerase chain reaction (PCR) or next generation sequencing (NGS) of cell-free DNA (cfDNA) in patient blood has begun to be applied to detect pathogen nucleic acids as a means to identify an infecting agent^5–7^.

cfDNA in humans is predominantly derived from human cells. It circulates in biological fluids (e.g., blood, urine) as a result of cellular apoptosis and necrosis^8,9^, with variable abundance (mean of ~10 pg/µL, range of 1–1000 pg/µL)^10^. It is also highly fragmented with the fragmentation size determined by the nucleosome-level organization of chromatin (peak fragment size of ~160 bp)^10–12^. A much smaller fraction of cfDNA is microbial in origin (< 1%), and its fragmentation size is much less well characterized but likely smaller than host nuclear cfDNA, since it lacks the same organization and protection lent by the mammalian nucleosome structure^13,14^. Nevertheless, fragments of cfDNA originating from pathogens at various body sites have been detected in purified plasma, prompting explorations of detecting these fragments for diagnosis^15^.

To achieve sensitive detection of low abundance, highly fragmented nucleic acids as would be required for bacterial cfDNA-based diagnostics, we took advantage of the attomolar sensitivity of SHERLOCK (Specific High Sensitivity Enzymatic Reporter UnLOCKing), a recently reported method applying nucleic acid detection to diagnostics. SHERLOCK combines traditional amplification with CRISPR/Cas13 detection, wherein amplified DNA is transcribed into RNA, and recognized by a complementary guide RNA (crRNA) complexed with the Cas13 enzyme; this interaction triggers collateral, non-specific Cas13 ribonuclease activity that is leveraged to generate a detectable reporter signal^16,17^. The amplification and detection steps provide two independent recognition steps to ensure high specificity. Collateral cleavage also results in signal amplification at the second step, so that both sensitivity and specificity are improved. Its sensitivity and specificity, as well as its requirement of only a relatively short recognition sequence of 28 nucleotides (nt) for the second recognition and detection step^18^, thus make it ideal for this application.

We recently adapted SHERLOCK to a microfluidic platform (DropArray) that enables the detection of comprehensive panels of viral pathogens (in a system called CARMEN; Combinatorial Arrayed Reactions for Multiplexed Evaluation of Nucleic acids) and bacterial pathogens (in a system called bCARMEN) in thousands of parallel nanodroplets, each containing the reagents for the unique detection of a single genetic locus of each pathogen^19,20^. However, given that the abundance of bacterial nucleic acids in blood cfDNA during infection may be well below one genome equivalent per reasonably collected volume of sample (50–5000 µL), the sensitivity of even the most sensitive assay will be limited by the frequency with which the target sequence is present in a queried sample. Splitting the initial collected sample into numerous parallel sub-samples for individual amplification or detection of different targets would necessarily reduce overall assay sensitivity. Meanwhile, detection of a single target alone could also contribute to suboptimal sensitivity by failing to take advantage of the fact that detection of any unique part of the fragmented bacterial genome would be sufficient for diagnosis. Detection of multiple targets would also ensure against mutations in any single target that could cause the assay to fail.

**Fig. 1:**
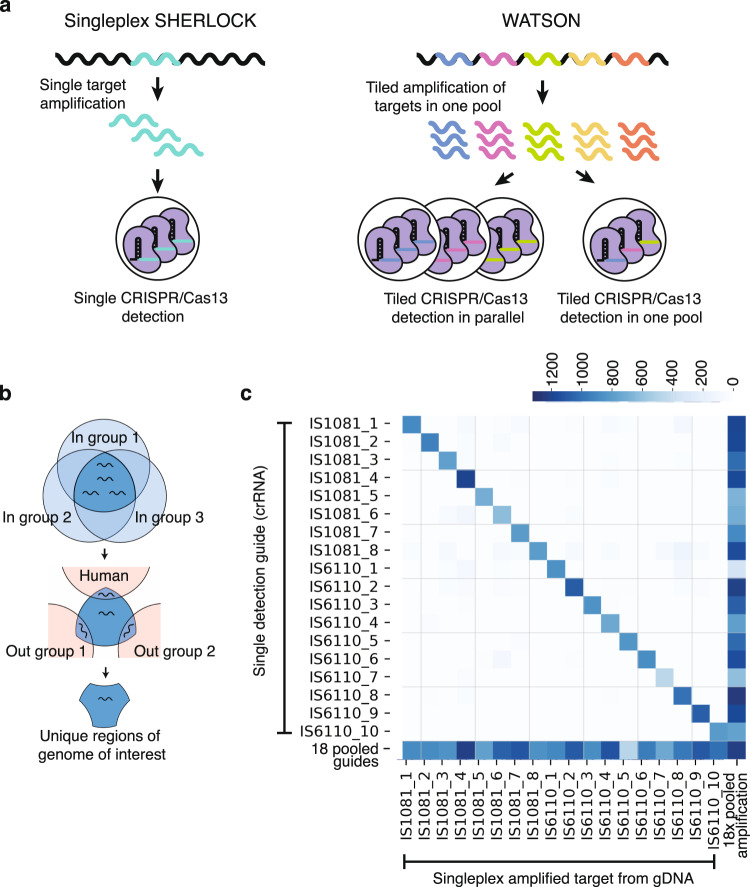
Experimental and computational workflow of WATSON. **a** Schematic of singleplex SHERLOCK and WATSON workflows; in singleplex SHERLOCK, a single genomic target is amplified, and this amplicon is detected by a single crRNA in a CRISPR/Cas13 reaction; in WATSON, tiled genomic targets are amplified in one pool, and amplicons are detected by CRISPR/Cas13 either in parallel amplicon-specific reactions or in one pooled reaction. **b** Computational workflow for identifying unique regions of the pathogen of interest; regions conserved across strains within the species (“In” group) are first identified; then regions present in related species (“Out” group) are excluded **c** Heatmap showing individual and pooled crRNA performance (detection; Y axis) with singleplex and 18-plex pooled amplification (amplification; X axis) of Mtb genomic DNA using DropArray^19^ at a fixed concentration of 10^3^ genome equivalents per reaction. Source data are provided as a Source Data file.

Here we developed a method, which we named WATSON (Whole-genome Assay using Tiled Surveillance Of Nucleic acids) to maximize the sensitivity of SHERLOCK for detecting cfDNA. We adapted SHERLOCK to perform pooled amplification followed by simultaneous detection of many target sequences tiled across the pathogen genome. (Fig. 1a) When a pathogen genome is present at concentrations less than one genome equivalent, some genomic loci may be present and others absent. By going after multiple genomic targets, we increase the odds of detecting at least one target in a sample, such that a pathogen can be deemed to be present as long as any one of those sequences is detected in a sample. This enables pathogen detection even far below the limit of a single genome equivalent per sample. The tiled detection step can be performed either by numerous parallel CRISPR/Cas13 detection reactions or by a single pooled detection reaction (Fig. 1a). Specifically, we applied WATSON to detect cfDNA from Mycobacterium *tuberculosis*, the causative agent of tuberculosis, a disease for which current diagnostic tests are highly dependent on the acquisition of pathogen-containing sputum from patients who are often unable to produce a high quality sample. As such, the WHO has prioritized the development of a rapid biomarker-based, non-sputum-based test to detect all forms of tuberculosis^21^. We demonstrate that WATSON not only has higher sensitivity than singleplex SHERLOCK (targeting a single locus) in engineered samples, but also importantly, that the tiling amplification and detection strategy can detect pathogen cfDNA in patients with active pulmonary tuberculosis. Finally, we also show the potential for translating WATSON to a field deployable, lateral flow platform, given the real-world requirements for diagnostics against infectious diseases such as tuberculosis.

## Results

### Computational design of tiled assay for detection of *M. tuberculosis* (Mtb) genomic sequences

We began by defining all possible sequence targets in the Mtb genome that are conserved across sequenced isolates of Mtb but are absent from related pathogens and the human genome (Fig. 1b). Because CRISPR/Cas13 detection requires a 28 nucleotide target sequence, we first computationally fragmented the Mtb strain H37Rv reference genome into all possible overlapping 28-mers, and then identified those 28-mers that were conserved across 267 whole, closed genome sequences of the Mycobacterium *tuberculosis* complex (MTBC) in the NCBI database, spanning its 7 human-adapted phylogenetic lineages^22^. Conservation was defined as no more than one single nucleotide polymorphism (SNP) in the 28-mer across all MTBC genomes. We found that 77% of all possible 28-mers in the reference genome were conserved across all 267 genomes. To then determine which of these conserved 28-mers were unique to MTBC, we aligned them to the genomes of 88 non-tuberculous mycobacterial (NTM) isolates covering over 20 species (Supplemental Data), reasoning that a MTBC-specific cfDNA assay was most likely to cross react with a closely related species. We also aligned the 28-mers to a reference human genome (GRCh38.p11 [https://www.ncbi.nlm.nih.gov/assembly/GCF_000001405.40]). Any 28-mers that differed by fewer than four SNPs from any of the 88 NTMs or human genomes were considered as not unique to MTBC and were excluded. The resulting 28-mers (65%) were considered to be both conserved across and unique to MTBC; they collectively spanned 79% of the reference genome. This conserved and unique part of the reference genome was used for primer and crRNA design.

To maximize assay sensitivity for proof of principle, we targeted multicopy genetic elements to increase the likelihood that any particular target is present in a sample containing highly fragmented, lowly abundant cfDNA. Specifically, we targeted the repetitive insertion elements IS6110 (1–25 copies per genome^23^, 16 copies in H37Rv) and IS1081 (5–7 copies per genome^24^, 6 copies in H37Rv). Unlike other TB NATs that also target IS6110 and/or IS1081, WATSON achieves a level of coverage across both repeat elements that is significantly greater than these other assays by virtue of tiling (our 18-plex assay covers 59% of the repeat elements, whereas other targeted assays cover only about 5%). Importantly, our computational workflow for primer and crRNA design is generalizable to tile across entire pathogen genomes, beyond targets like the IS6110 and IS1081 elements, which we demonstrate in this study as proof of principle. To facilitate the pooling of primers in the first amplification step, primers were designed to minimize 3’ − 3’ interactions by ensuring that the 5 nucleotides at the 3’ terminal end of each primer did not complement any part of any of the other primers in the pool (see Methods). A T7 promoter sequence was appended on the 5’-end of one primer of each pair to allow the amplified product to be transcribed into RNA for CRISPR/Cas13 detection. We identified 18 primer pairs that spanned 18 non-overlapping regions within these elements and contained at least one of the computationally-defined 28-mers described above, corresponding to a crRNA target sequence. Some amplicons contained several adjacent overlapping 28-mers, which provides flexibility in crRNA design. The amplicons ranged in size from 70 to 97 bp, with the gap between the forward and reverse primers ranging from 28 to 48 bps, and collectively spanned 58% of the IS6110 and IS1081 sequences.

We then generated crRNA for the Cas13-based detection step corresponding to the 28-mers contained within each of the 18 amplicons. If an amplicon allowed for adjacent overlapping 28-mers, we designed and tested up to three crRNA for each target amplicon. crRNA were tested with singleplex and 18-plex PCR amplified Mtb gDNA using the previously reported DropArray microfluidic platform^20,25^. From among 29 crRNA tested, we chose the best 18 crRNA spanning the 18 primer pairs to be included in a WATSON assay based on their ability to produce a strong positive signal (> 6 standard deviations above the no template control sample) when tested against Mtb genomic DNA both in singleplex and 18-plex pooled format (Fig. S1, Fig. 1c). Finally, we performed a BLAST search of all 18 pairs of primers and crRNA against all prokaryotes in NCBI and confirmed that they did not have significant homology (E-value < 1) with any non-MTBC pathogens. Primer and crRNA sequences are listed in Table S1.

### Evaluation of WATSON on engineered samples

We first evaluated the performance of WATSON using fragmented gDNA as input material. Purified gDNA from Mtb H37Rv was enzymatically fragmented to a median size of 180 bp^5,26,27^ (Fig. S2). We then created a dilution series of the fragmented Mtb gDNA amidst a constant background of 1 genome equivalent (GE) per µL of purified, fragmented human gDNA, to mimic what has been reported physiologically for human plasma^10,12^. We performed pooled 18-plex amplification followed by CRISPR/Cas13 detection in nanodroplets using the DropArray platform^19,20^, either in parallel with each individual crRNA, or in a single pool of all 18 crRNAs together.

**Fig. 2:**
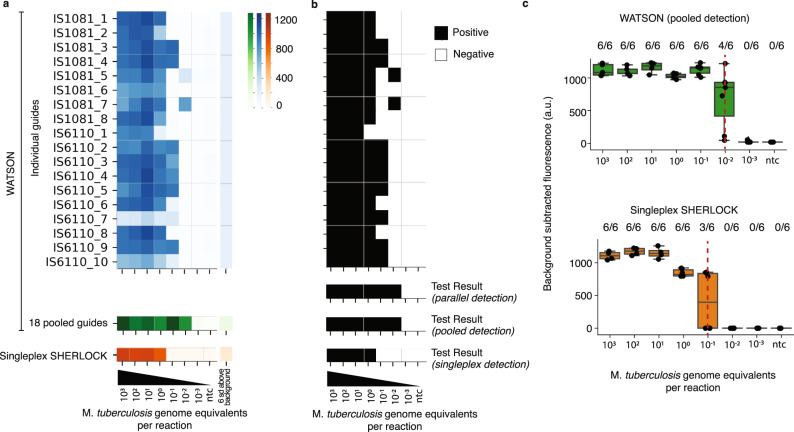
Evaluation of WATSON limit of detection (LoD) on engineered samples. **a** Example of WATSON and singleplex SHERLOCK fluorescence levels across a dilution series of fragmented Mtb gDNA; heatmap of WATSON signals are shown for each individual crRNA tested in parallel (blue) as well as in a single pool of 18 crRNAs (green); singleplex SHERLOCK signal using a primer pair and crRNA targeting IS6110_2 (orange) **b** Binary calls. A positive call from any one of these 18 independent signals yields a positive Test Result (parallel detection). A binary call of the 18-pooled crRNA signals is shown as Test Result (pooled detection). **c** WATSON (pooled detection; top) and singleplex SHERLOCK (bottom) LoD determined from six replicate reactions at each dilution. The line in the center of each box plot represents the median value based on the six replicates tested at each dilution of fragmented Mtb genomic DNA, where the bounds of the box span the interquartile range of observations, and the top and bottom whiskers represent the samples with the maximum and minimum background subtracted fluorescence value of the six replicates, respectively. The numbers above each box plot are the fraction of replicates for which the fluorescence signal was > 6 standard deviations above the no template control sample. LoD is shown by the red dotted line where variable signals are observed across replicates; NTC = no template control. Source data are provided as a Source Data file.

A heatmap for an exemplary dilution series shows fluorescent signals from each CRISPR/Cas13 detection reaction, generated by CRISPR/Cas13 collateral cleavage of a fluorescent reporter, for each individual crRNA tested in parallel and provides information on which of the individual tiled targets is present in the sample (Fig. 2a). The heatmap signals can be converted to a binary call of positivity based on a value > 6 standard deviations above the average fluorescence of the no template control sample (Fig. 2b). A positive signal from any one of these 18 independent readouts is sufficient to yield a positive test result determination, reflecting the principle that the detection of any one target sequence is sufficient for a positive result (Fig. 2b). Similarly, the fluorescent signal from a single, pooled CRISPR/Cas13 detection reaction can be converted to a binary test result based on a similar threshold of > 6 standard deviations above the no template control. The test results from parallel and pooled detection were the same, detecting Mtb down to an input of 0.01 GE per reaction (Fig. 2b). While parallel detection provides detailed information on the performance of individual guides, pooled detection makes the assay as technically simple as possible.

To compare the limit of detection (LoD) of WATSON (pooled amplification and pooled detection) to singleplex SHERLOCK (singleplex amplification and detection), we performed six replicates of the dilution series with each assay. For singleplex SHERLOCK, the single best primer pair and crRNA from this set (IS6110_2) was used. Singleplex SHERLOCK had a LoD between 0.1 and 1 Mtb GE/reaction (0.5–5 fg of fragmented DNA), while WATSON using pooled detection showed an improvement in LoD by 10- to 100-fold (0.01–0.1 GE/reaction, Fig. 2c). Variable signal across replicates at the LoD is consistent with a stochastic distribution of fragments in the samples at low copy numbers (Fig. S3). WATSON showed a comparable detection signal when testing DNA from Mtb strains across the phylogenetic tree, although LoD varied depending on the expected number of copies of IS6110 in the strain (Fig. S4). WATSON did not produce a detectable signal when DNA from other NTMs and other clinically relevant bacteria were tested (Fig. S5).

### Evaluation of WATSON on clinical samples

Having determined the sensitivity and specificity of WATSON in engineered samples of the reference strain, we then sought to address WATSON’s ability to detect Mtb nucleic acids in cfDNA of patients with tuberculosis and compare tiling to detection of a single locus. We compared the 18-plex version of WATSON to singleplex SHERLOCK on clinical plasma samples obtained from patients with active pulmonary tuberculosis as confirmed by sputum-based culture and/or the Cepheid GeneXpert qPCR diagnostic test.

**Fig. 3:**
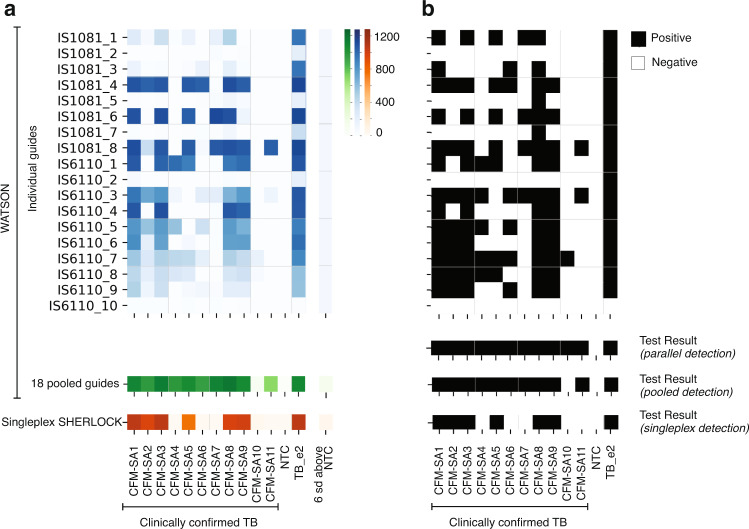
Comparison of WATSON and singleplex SHERLOCK on clinical samples from South Africa. cfDNA extracted from the equivalent of 400 µL of patient plasma was amplified in separate experiments by either a pool of 18 primers (WATSON) or a single primer pair (Singleplex SHERLOCK). **a** Heatmap of WATSON fluorescence signals for 11 clinical samples are shown for each individual crRNA tested in parallel (blue) as well as in a single pool of 18 crRNAs (green); singleplex SHERLOCK signal using a crRNA targeting IS6110_9, which coincides with the same region of IS6110 as the cfDNA-based qPCR assay that was also used to evaluate these samples (IS6110_9) (orange). The fluorescent signal corresponding to 6 standard deviations above the average fluorescence of the no template control samples is shown as a visual representation of the analysis performed to convert the fluorescent signals in (**a**) to the binary test results shown in (**b**). **b** Fluorescent signals converted to binary Test Result based on a cut-off signal > 6 standard deviations above the no template control. (CFM-SA = Confirmed TB patient from South African cohort that tested positive by culture and GeneXpert; NTC = no template control; TB_e2 = positive control where fragmented gDNA from Mtb H37Rv at 10^2^ GE/reaction was used as the template). Source data are provided as a Source Data file.

We started with clinical samples from South Africa that were also positive for blood cfDNA as confirmed by a cfDNA-based qPCR assay that targets a single 72 bp region of IS6110^28^. (Table 1) cfDNA was extracted from the equivalent of 400 uL of plasma from 11 patients. WATSON detected a positive signal in 10 of the 11 samples, positively identifying 91% on this small set of samples while singleplex SHERLOCK was positive in only 6 of the 11 samples (55%), highlighting the improved performance and thus value of tiling over single locus targeting. (Fig. 3) (The single difference observed between singleplex SHERLOCK and the qPCR assay for some samples was likely due to differences in numbers of thermal cycles used for amplification for the various assays (see Methods)).

**Table 1:** Metadata, tuberculosis (TB) tests performed, and TB diagnosis for clinical samples from South Africa

Clinical metadata			Diagnostic test for TB				TB diagnosis		
Sample ID	Region	Clinical status	Culture*	GeneXpert*	qPCR**	WATSON***	Confirmed active pulmonary TB^†^	WATSON^††^	SHERLOCK
CFM-SA1	South Africa	Respiratory illness	+	+	34.0	13/18	+	+	+
CFM-SA2	South Africa	Respiratory illness	+	+	39.0	8/18	+	+	+
CFM-SA3	South Africa	Respiratory illness	+	+	35.6	12/18	+	+	+
CFM-SA4	South Africa	Respiratory illness	+	+	40.1	5/18	+	+	−
CFM-SA5	South Africa	Respiratory illness	+	+	37.9	7/18	+	+	+
CFM-SA6	South Africa	Respiratory illness	+	+	38.4	6/18	+	+	−
CFM-SA7	South Africa	Respiratory illness	+	+	40.9	4/18	+	+	−
CFM-SA8	South Africa	Respiratory illness	+	+	37.1	15/18	+	+	+
CFM-SA9	South Africa	Respiratory illness	+	+	38.2	11/18	+	+	+
CFM-SA10	South Africa	Respiratory illness	+	+	38.7	1/18	+	−	−
CFM-SA11	South Africa	Respiratory illness	+	+	40.6	2/18	+	+	−

*CFM-SA* Confirmed TB patient from South African cohort who tested positive by culture and GeneXpert, + = TB-positive, − = TB-negative

*Sputum-based culture and/or GeneXpert are WHO-recognized TB tests and were used as the reference standard and comparator test, respectively, to determine the TB status of the participants who presented with a respiratory illness.

**A cfDNA-based qPCR assay was performed in this study as a measure of amplifiable cfDNA. Shown are the Ct values. A “Negative” qPCR result reflects a sample where amplification did not cross the assay cut-off threshold.

***WATSON using parallel detection, with the fluorescent signal from each of 18 crRNA converted to a binary test result, was performed in this study. Shown are the number of crRNA that produced a signal > 6 standard deviations above the no template control sample.

^†^TB status determined based on the results from culture and GeneXpert testing.

^††^WATSON results based on pooled detection.

We next tested WATSON on a broader set of clinical plasma samples from Uganda that included 9 patients with active pulmonary tuberculosis, as confirmed by sputum culture and/or GeneXpert but which had not been pre-screened by qPCR, 6 clinically suspected but sputum culture- and GeneXpert- negative cases, and 26 healthy controls. We thus also performed qPCR targeting the single region of IS6110 on the same cfDNA extracted from the samples to compare methods and evaluate the benefits of tiling^28^ (Table 2).

**Table 2:** Metadata, tuberculosis (TB) tests performed, and TB diagnosis for clinical samples from Ugandan and American cohorts

Clinical metadata			Diagnostic test for TB				TB diagnosis	
Sample ID	Region	Clinical status	Culture*	GeneXpert*	qPCR**	WATSON ***	Confirmed active pulmonary TB†	WATSON
HC1-6 (6 samples)	Palo Alto, CA	Healthy	ND (all 6 samples)	ND (all 6 samples)	Negative (all 6 samples)	0/18 (all 6 samples)	− (all 6 samples)	− (all 6 samples)
HC7-26 (20 samples)	Watertown, MA	Healthy	ND (all 20 samples)	ND (all 20 samples)	ND (all 20 samples)	0/18 (all 20 samples)	− (all 20 samples)	− (all 20 samples)
SUS1	Uganda	Respiratory illness	−	−	Negative	0/18	−	−
SUS2	Uganda	Respiratory illness	−	−	Negative	0/18	−	−
SUS3	Uganda	Respiratory illness	−	−	Negative	0/18	−	−
SUS4	Uganda	Respiratory illness	−	−	Negative	0/18	−	−
SUS5	Uganda	Respiratory illness	−	−	Negative	4/18	−	+
SUS6	Uganda	Respiratory illness	−	−	Negative	2/18	−	+
CFM-UP1	Uganda	Respiratory illness	ND	+	36.1	15/18	+	+
CFM-UP2	Uganda	Respiratory illness	ND	+	33.9	15/18	+	+
CFM-UP3	Uganda	Respiratory illness	ND	+	36.1	15/18	+	+
CFM-UP4	Uganda	Respiratory illness	ND	+	39	16/18	+	+
CFM-UP5	Uganda	Respiratory illness	ND	+	35.8	16/18	+	+
CFM-UP6	Uganda	Respiratory illness	ND	+	38.5	16/18	+	+
CFM-UP7	Palo Alto, CA	Respiratory illness	+	+	31.5	9/18	+	+
CFM-UP8	Palo Alto, CA	Respiratory illness	+	+	Negative	2/18	+	+
CFM-UP9	Palo Alto, CA	Respiratory illness	+	+	Negative	0/18	+	−

*HC* Healthy control, *SUS* Suspected TB patient who presented with a cough for more than a week but tested negative by culture and GeneXpert, *CFM-UP* Confirmed TB patient from Ugandan or Palo Alto cohort who tested positive by culture and/or GeneXpert, *ND* Not done, + = TB-positive, − = TB-negative

*Sputum-based culture and/or GeneXpert are WHO-recognized TB tests and were used as the reference standard and comparator test, respectively, to determine the TB status of the participants who presented with a respiratory illness.

**A cfDNA-based qPCR assay was performed in this study as a measure of amplifiable cfDNA. Shown are the Ct values. A “Negative” qPCR result reflects a sample where amplification did not cross the assay cut-off threshold.

***WATSON using parallel detection, with the fluorescent signal from each of 18 crRNA converted to a binary test result, was performed in this study. Shown are the number of crRNA that produced a signal > 6 standard deviations above the no template control sample.

^†^TB status determined based on the results from culture and/or GeneXpert testing.

**Fig. 4:**
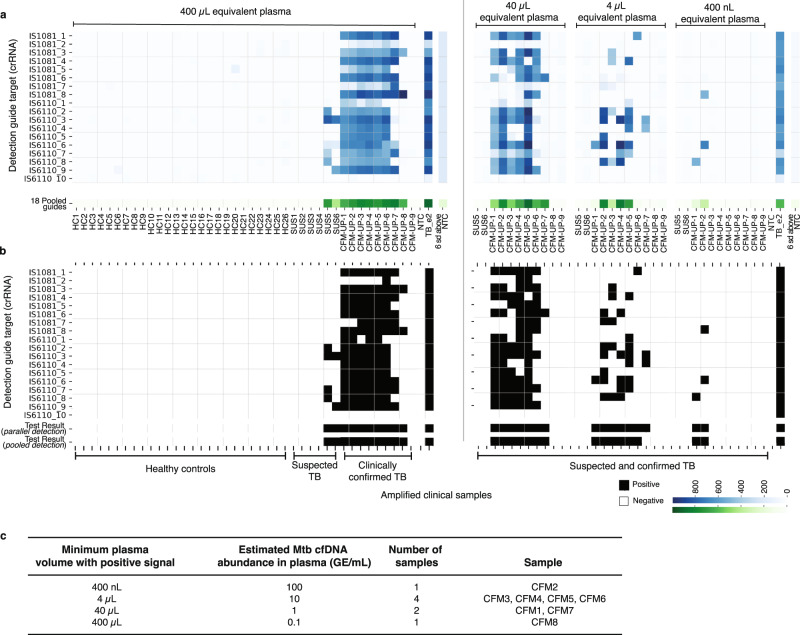
Evaluation of WATSON on clinical samples. cfDNA extracted from the equivalent of 400 µL of patient plasma was amplified by a single pool of 18 primer sets. **a** Heatmap of WATSON fluorescence for 41 clinical samples for each crRNA tested in parallel (blue) and for all 18 crRNA pooled together in a single reaction (green). For positive samples, a series of 10-fold dilutions of extracted cfDNA was tested; the equivalent volume of plasma from which the amount of cfDNA in each dilution is extracted is indicated above. The fluorescent signal corresponding to 6 standard deviations above the average fluorescence of the no template control samples is shown as a visual representation of the analysis performed to convert the fluorescent signals in (**a**) to the binary test results shown in (**b**). (HC Healthy control, SUS Suspected TB patient who presented with a cough for more than a week but tested negative by culture and GeneXpert, CFM-UP = Confirmed TB patient from Ugandan or Palo Alto cohort that tested positive by culture and/or GeneXpert). **b** Fluorescent signals converted to binary Test Result based on a cut-off signal (> 6 standard deviations above the no template control sample). **c** Summary of estimated cfDNA abundance in confirmed tuberculosis samples based on the minimum plasma volume needed to observe a positive signal by any of the eight IS1081 crRNAs. Source data are provided as a Source Data file.

We first evaluated WATSON on cfDNA extracted from the equivalent of 400 µL of patient plasma and detected a positive signal in 8 of 9 samples (89%) from confirmed active tuberculosis patients (CFM-UP1-9) and in 0 of 26 healthy controls (HC1-26, Fig. 4a). In 6 of the 8 confirmed TB-positive samples (CFM-UP1-6), nearly all (> 14/18) crRNAs individually produced a signal. In one sample (CFM-UP7), 9/18 crRNAs produced a signal. In the other positive sample (CFM-UP8), only 2/18 crRNAs produced a positive signal, suggesting a very low abundance of Mtb cfDNA. Of note, 2 of the 9 WATSON-positive samples were qPCR-negative (CFM-UP8-9) highlighting WATSON’s potential to detect Mtb cfDNA over the singleplex qPCR assay. Additionally, we tested 400 µL cfDNA samples from 6 sputum culture-, GeneXpert-negative, but clinically suspected tuberculosis patients (SUS1-6); all 6 were also negative by qPCR. However, interestingly, in 2 of the 6 samples from suspected tuberculosis cases that lacked laboratory confirmation by any other method, a positive signal was detected, albeit from a minority of targets (4/18 in SUS5 and 2/18 in SUS6). Unfortunately, no clinical follow-up was available for the two patients from which these two samples were collected.

We then retested all 9 samples from patients confirmed to have active pulmonary TB and the 2 samples from suspected TB patients that were WATSON-positive (CFM-UP1-9 and SUS5,6) using limiting amounts of sample input volume (cfDNA extracted from the equivalent of 40 µL, 4 µL and 400 nL of plasma). Unsurprisingly, the three positive samples in which only a minority of targets were detected from the equivalent of 400 µL (CFM-UP8, SUS5 and SUS6) were negative when the input volume was decreased 10-fold (i.e., 40 µL). In contrast, the remaining 7 positive samples were positive not only when 40 µL was used, but even when the equivalent of just 4 µL of plasma was used as the input, with two samples being positive even with an input equivalent to as little as 400 nL of plasma. Parallel detection and pooled detection were highly concordant, with 43/44 calls in agreement across the 4 input levels of these 11 samples, with a single discrepancy (CFM-UP7 at 4 µL).

With a small input volume (the equivalent of 4 µL of plasma), WATSON detected a positive signal in 7 of the 9 confirmed positive samples (78%) (Fig. 4b, parallel detection). Importantly, this level of detection is the direct result of the increased opportunities for target detection provided by tiling, since no individual crRNA was detected in more than 44% of these samples. Additionally, and importantly, these results confirmed the presence of Mtb cfDNA fragment sizes that are compatible with WATSON’s amplification and detection strategy. Intriguingly, WATSON’s detection of Mtb gDNA in two suspected tuberculosis cases that lacked laboratory confirmation raises the tantalizing possibility that WATSON might be able to detect cases which current diagnostic approaches miss; additional follow up would be required to understand this possibility.

We sought to estimate the amount of Mtb cfDNA in the plasma samples of patients with active pulmonary tuberculosis using WATSON. Since IS6110 copy number can vary widely across Mtb strains^29^ (from 1 to 25), only signals produced by IS1081 were used for these estimates. Based on the data from our engineered samples, WATSON’s LoD using only IS1081-based amplicons is ~0.05 GEs/reaction. Using this LoD as a benchmark and the minimum sample volume producing a positive signal from a given patient plasma sample, we back-calculated the estimated amount of Mtb cfDNA in the original sample. Efficiency of cfDNA extraction from plasma was assumed to be on the order of magnitude of 100%, based on previously reported data for the method used in this study^30^. 1 of 9 positive samples (11%) had on the order of 100 GE/mL, 4 of 9 samples (44%) had 10 GE/mL, 2 of 9 samples (22%) had 1 GE/mL, and 1 of 9 samples (11%) had approximately 0.1 GE/mL. This suggests a very wide dynamic range (over 3 orders of magnitude) in Mtb cfDNA abundance in confirmed pulmonary tuberculosis patients (Fig. 4c).

### Potential for a point-of-care diagnostic workflow

As a first step towards demonstrating the potential for moving WATSON onto a point-of-care platform with progress towards addressing some of the most infrastructure-heavy aspects, we demonstrated the ability to detect Mtb cfDNA using isothermal recombinase polymerization amplification (RPA) to replace PCR thermocycling, and a lateral flow strip to replace fluorescence Cas13 signal detection^16,31^.

**Fig. 5:**
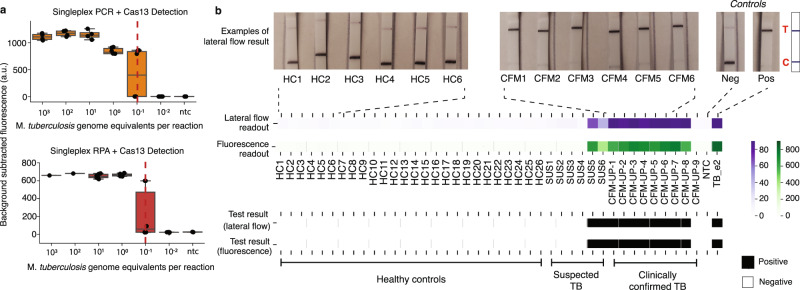
Progress towards a field deployable assay format using RPA and lateral flow readout. **a** Singleplex PCR (orange) and RPA (red) amplification using a primer pair targeting IS6110_2 combined with CRISPR/Cas13 detection show similar limits of detection (indicated by dotted red line). The line in the center of each box plot represents the median value based on the six replicates tested at each dilution of fragmented Mtb genomic DNA, where the bounds of the box span the interquartile range of observations, and the top and bottom whiskers represent the samples with the maximum and minimum background-subtracted fluorescence value of the six replicates, respectively. **b** WATSON on cfDNA extracted from the equivalent of 400 µL of patient plasma samples using pooled detection, evaluated using either lateral flow readout from image analysis of lateral flow strip (purple) or fluorescence readout (green); fluorescence (RFU) and Lateral flow readouts are converted to a binary Test Result (positive = black; negative = white) based on a cut-off (> 6 standard deviations above the no template control sample). (HC = Healthy Control, SUS = Suspected TB Patients who presented with a cough for more than a week but tested negative by culture and GeneXpert, CFM-UP = Confirmed TB patient from Ugandan or Palo Alto cohort who tested positive by culture and/or GeneXpert). Representative lateral flow strips from negative and positive clinical samples as well as controls are shown above. A schematic of the lateral flow strip is shown to the right of the images where ‘T’ is the test line and ‘C’ is the control line (Fig. S6). More images of lateral flow strips are shown in Fig. S7. Source data are provided as a Source Data file.

In the original description of SHERLOCK, RPA was used as the amplification method, favored for its isothermal nature, which dispenses with thermal cycling. We screened over 20 RPA primer pairs and selected the primer/crRNA combination with the best LoD with engineered samples. Importantly, this pair, targeting a single 89 bp region of the multicopy IS6110 element, had a sensitivity comparable to that of singleplex PCR (Pearson’s *R* = 0.91), when coupled with CRISPR/Cas13 detection, suggesting that moving from PCR to RPA amplification will likely also ultimately have the needed sensitivity required for bacterial cfDNA detection. While RPA cannot currently be multiplexed as widely as PCR, it has several advantages for point-of-care deployment, most notably isothermal amplification at temperatures achievable without specialized equipment, thus motivating ongoing efforts to achieve highly multiplexed RPA (Fig. 5a).

Another key aspect of point-of-care testing is ease of signal detection. The collateral RNAse activity of the activated Cas13 enzyme can be exploited in a variety of ways to provide an easily interpretable diagnostic result. By placing a Cas13-cleavable RNA linker between biotin and fluorescein. Cas13 activation has been shown to be detectable in a lateral flow assay (LFA)^31^, a convenient format for field-deployable, point-of-care tests in resource-limited settings (Figure S6). To evaluate LFA sensitivity for cfDNA from plasma of patients with active pulmonary tuberculosis, we compared the detection of LFA signal, quantified by image analysis (see Methods), with fluorescence signal for the same set of 18-plex pooled PCR amplified clinical samples. We found the two detection modalities to be concordant - affording the same sensitivity and specificity of assay for the equivalent of 400 µL patient plasma. We thus demonstrate Mtb cfDNA detection in a LFA platform and a path forward for its potential development as a point-of-care test (Fig. 5b, S7).

## Discussion

Liquid biopsies are beginning to revolutionize disease diagnostics and management^8,32,33^. In particular, detection of circulating microbial cfDNA has the potential to transform infectious disease diagnostics given its sensitivity, relatively non-invasive nature, applicability to many different infection types, and potential robustness to prior, recent antibiotic treatment. Indeed, there is growing clinical evidence on the efficacy of NGS-based microbial cfDNA tests^32,34^. The advantage of sequencing microbial cfDNA is its sensitivity in detecting rare pathogen nucleic acids, since the detection of any fragment of the pathogen genome may be sufficient for diagnosis and de novo sequencing requires no hypothesis about organism identity, although in practice the presence of the pathogen in a reference database, a minimum genome coverage threshold or other metrics may be needed to improve specificity^6^. Offsetting this advantage is the potential loss in sensitivity due to complex library construction, wherein short and single stranded fragments may be lost^12^. Additional key disadvantages with NGS are the significant infrastructure, cost, and time currently needed to process and sequence samples, and interpret results. As an alternative, PCR-based detection of cfDNA has been proposed, but is not yet clinically deployed due, in large part, to insufficient sensitivity of these assays as current PCR-based cfDNA assays typically only detect a single genomic sequence. For example, in the case of Mtb, most of the reported sensitivities range from 45% to 65%^35–37^. Encouragingly, a recent study reported improved sensitivity (> 90%) in a pooled adult and pediatric group using a singleplex CRISPR-based diagnostic, albeit on limited sample numbers from a single geographical location^7^. Here we demonstrate that tiling – pooled amplification of targets across the genome with pooled CRISPR/Cas13-based detection of these targets - affords improved sensitivity over singleplex assays, which will pave the way for improving diagnostic sensitivities even further and minimizing the volume of blood that is required from patients.

In this study, we developed WATSON, a highly sensitive and specific assay that combines tiled, pooled amplification and CRISPR/Cas-13 detection that is able to detect cfDNA in patients with active pulmonary tuberculosis, with progress toward a potentially field-deployable, point-of-care platform. We have created a comprehensive assay development workflow for WATSON as a modular diagnostic, starting with computational design of tiled amplification primers and crRNA that takes into account pathogen genomic diversity, testing and validation of primer and crRNA pools, to a final implementation in a single pooled amplification and detection step. By taking a tiled approach that can detect relatively short genomic fragments, we increase the likelihood of detecting any one target that is present in a sample, thereby enabling our approach to detect considerably less than 1 genome equivalent per sample. Additionally, we demonstrate that this CRISPR/Cas-13 detection strategy is able to accommodate the very short fragments of pathogen nucleic acids present in patient blood as cfDNA. WATSON thus has the potential to be applied to the detection of pathogen cfDNA as a new approach to infectious disease diagnostic testing and as a tool that enables the study of the microbial cfDNA landscape in the context of infection. As a liquid biopsy approach, it has the potential to obviate the need for more invasive sampling of some infections and make sample collection more uniform for all infection-types.

In this study, we found that WATSON detected Mtb cfDNA in 10 of 11 samples (91%) obtained from confirmed TB patients from the South African cohort and 8 of 9 samples (89%) from confirmed TB patients from the Ugandan cohort (Tables 1, 2). This performance was achieved using cfDNA from 400 µL of plasma, a volume similar to or less than previous studies. Even when using 100-fold less input volume (equivalent of 4 µL plasma), WATSON was able to detect Mtb cfDNA in 7 of 9 TB-confirmed samples (78%). This work reveals the wide range in abundance of Mtb cfDNA present in confirmed active pulmonary tuberculosis patients. It also highlights the potential of WATSON. Clearly more samples will need to be tested to truly understand the clinical sensitivity of WATSON across a wide geographical distribution of patients with tuberculosis and thus wide phylogenetic distribution of strains. However, if the need for higher sensitivity is recognized as more samples are tested, this can be achieved by WATSON not only by collecting and testing larger volumes of blood, but importantly, by increasing tiling across more of the genome. There are many more loci within the entire Mtb genome that could be additionally targeted beyond this initial proof of principle set of 18 primer pairs and crRNA that currently targets only the IS6110 and IS1081 regions of the Mtb genome. Conversely, if increased tiling across many more targets can drive the sensitivity to even lower LoDs, then only very small amounts of collected blood will be required. To further address the need for a rapid, point-of-care diagnostic, we demonstrated the potential for the workflow to be adapted to more point-of-care settings, using a lateral flow assay (LFA) and an isothermal method for amplification. Importantly, LFA sensitivity was comparable to fluorescence readout, and singleplex RPA was comparable to singleplex PCR. The goal of this work was to demonstrate that WATSON is able to detect cfDNA in patients; further work will be needed to optimize sensitivity, enable tiled isothermal amplification of multiple genomic targets, and convert the entire workflow, including cfDNA extraction to be compatible with whole blood, from end to end into a field-deployable format. However, this work demonstrates a path toward a fully-integrated test that can be deployed in resource-limited settings where infrastructure-heavy molecular diagnostics, such as qPCR and next generation sequencing, are currently not feasible.

While WATSON showed a 10- to 100-fold improvement in analytical sensitivity over singleplex SHERLOCK, the potential for WATSON to improve in clinical testing is highlighted in the 2 of 6 clinically suspected tuberculosis samples, despite their being negative by sputum-based culture and GeneXpert testing as well as cfDNA-based qPCR testing. It is tantalizing to think that the assay may be able to detect infections that currently elude current assays. More samples across multiple disease states, with clinical correlation, will need to be tested in order to learn how to interpret such results appropriately.

Given WATSON’s ability to detect Mtb cfDNA in blood, we believe it holds the potential to be used as a diagnostic for many different infectious syndromes or pathogens by detecting cfDNA of the infecting agent in blood as well as other body fluids such as urine, which has been shown to contain microbial cfDNA^38,39^. While its sensitivity for different pathogens in different infection types needs to be determined empirically, the programmable nature of nucleic acid detection has no limits with regards to the range of pathogens to which it could be applied. In addition to its application as a diagnostic, given the high test performance level, technical ease and minimally invasive nature of WATSON, it has the potential to be applied in other ways to impact patient care, including following circulating pathogen cfDNA levels as a biomarker to define the efficacy and even duration of antibiotic treatment^40^ or as an alternative metric for efficacy in clinical trials of drugs. In the case of Mtb, cfDNA levels could potentially supplement or even replace sputum-based measures of early bactericidal activity^41^, thereby informing drug development and other interventional strategies. However, much remains to be learned about microbial cfDNA as a biomarker including how microbial cfDNA distribution correlates with disease state (*i.e*., latent versus active tuberculosis, pulmonary versus extrapulmonary disease), the kinetics of clearance upon treatment, and how it tracks with disease progression or cure.

Here we present WATSON, a nucleic acid detection method that builds on existing CRISPR-diagnostics and represents a strategy for designing and detecting multiple genomic targets while remaining substantially faster and easier to execute than current sequencing-based tests. It leverages the two-step pre-amplification with detection by Cas13, to ensure the unique capability of detecting very short nucleic fragments with high sensitivity and specificity, and improves on existing cfDNA strategies through tiling across the pathogen genome. Importantly, this proof-of-concept study reveals that detectable DNA of pathogens such as Mtb can be found in the plasma of a high frequency of patients with pulmonary TB, and the power of tiling to improve our ability to detect it. With improved assays, detection of cfDNA for diagnosis of tuberculosis is increasingly feasible, with WATSON potentially applicable to a much broader range of infections.

## Methods

All methods used in this study complies with all relevant ethical regulations. Approval for the collection of clinical samples was obtained from the institutional review boards (IRB) at the Uganda National Council for Science and Technology or Stanford University.

### Identification of unique MTBC genomic regions

Computational analysis was done using custom Python scripts. Complete, closed genome sequences without gaps were used to identify suitable targets. They were downloaded from NCBI and included 267 whole genome sequences of the Mycobacterium *tuberculosis* complex (MTBC), 88 sequences from non-tuberculous mycobacteria (NTMs), and the reference human genome (GRCh38.p11 [https://www.ncbi.nlm.nih.gov/assembly/GCF_000001405.40]) The reference MTB sequence (H37Rv, accession: NC_000962) was broken down into a sliding set of 28-mers. The total number of 28-mers generated was 4,411,504 (i.e., the size of the genome minus 28). These 28-mers were then tested for alignment with all other MTBC sequences (‘in-group’), as well as NTMs and human genome (‘out-group’), using a fast sequence alignment tool (Bowtie2). 28-mers that were one or fewer SNPs apart across all MTBC sequences were defined as the conserved targets (77% of all targets). These 28-mers were then screened to exclude any that were less than 4 SNPs away from any part of the out-group genomes. The remaining targets (65% of original) were then mapped back to the reference genome (H37Rv) and these genomic regions were used for further analysis.

### Genome-wide tiled primer design

Effective multiplexing can be undermined by off-target interactions between primers. To minimize detrimental interactions among pooled primers, we designed primers based on the principle of minimizing 3’−3’ interactions between primer pairs. All primers in a pool contained the same set of 5-mers at their 3’ ends, where the sequence of compatible 5-mers and corresponding primer pairs were determined by an iterative, heuristic search algorithm. First, all possible 5-mers (4^5^ = 1024) were ranked based on how many times they were present in the top strand of the IS6110 and IS1081 regions. 5-mers that contained three or more repeated nucleotides were removed from this list of 5-mers. Of the remaining ranked 5-mers, we selected the top 100 and identified the loci of potential primer pairs for each one based on whether that 5-mer and its reverse complement were separated by 28 to 48 base pairs to allow for crRNA binding. To create a pool of primers wherein all primers contained the same 5-mer sequence at the 3’ end, we started with the top ranked 5-mer, generated 30 nucleotide-long primer pairs at all of the identified loci, and only included those in the pool if 1) amplicons were non-overlapping with any others in the pool, 2) neither forward or reverse primers contained a stretch of 5 bases complementary to the 5-mer and 3) they did not contain stretches of four or more homopolymers. The 5’-end of primers were then extended or trimmed to ensure their melting temperatures were within 63−65 ˚C. To increase the size of a pool, we allowed primers to contain a second 5-mer sequence at their 3’ ends – in addition to the 5-mer from the first round that yielded the greatest number of primers in a pool - by generating another set of 30 nucleotide-long primer pairs, each containing one of the two “allowed” 5-mer sequences and only included those in a pool if 1) amplicons were non-overlapping with any others in the pool, 2) neither forward or reverse primers contained a stretch of 5 bases complementary to either of the two 5-mers in the pool, and 3) they did not contain stretches of four or more homopolymers. We generated all possible pools for primers that contained up to 15 different 5-mer sequences at their 3’ ends; and identified a maximum of 18 primer pairs that could be pooled together, wherein each primer pair contained one of 11 different 5-mer sequences at their 3’ end. (Fig. S8).

### Primer and crRNA preparation

Individual primers were ordered from Integrated DNA Technologies and resuspended in nuclease-free water and stored at −20 ˚C. crRNAs were ordered as complementary ssDNA sequences with a T7 promoter binding sequence attached to the 5’-end. Each crRNA was synthesized via in vitro transcription (IVT) using the HiScribe T7 High Yield RNA Synthesis Kit (New England Biolabs) by incubating a ssDNA template (at 1 µM final concentration) in reaction buffer at 37 ˚C with T7 promoter primer (1 µM final concentration) for 12 h. In vitro transcribed product was then diluted down to a final concentration of 225 nM of crRNA and quantified using a Nanodrop instrument (Thermo Scientific). crRNAs were stored at −80 ˚C. For one crRNA, (IS6110_2), in addition to IVT, synthetic RNA was also purchased from Synthego Corporation. In this case, the RNA was rehydrated with nuclease free water, diluted to 225 nM, and stored at −80 ˚C. For pooled crRNA detection, all 18 crRNAs were mixed to a total concentration of 225 nM (12.5 nM per crRNA) and stored at −80˚C until further use. The sequences of all primers and crRNAs are provided in Table S1.

### Bacterial culture and genomic DNA preparation

Genomic DNA was isolated from Mycobacterium *tuberculosis* H37Rv grown in Middlebrook 7H9 medium supplemented with OADC using a cetrimide-based protocol as previously described^42^. *Mtb* genomic DNA (1 µg) was digested with NEBNext dsDNA Fragmentase (30 min for a median size of ~180 bp) using 2X the recommended concentration of Fragmentase enzyme. Fragmented gDNA was purified with AMPure XP DNA SPRI beads (2.5x) and eluted in 20 µL nuclease-free water. The concentration of purified fragmented DNA was quantified using Qubit (Life Technologies), and fragment size profiles were determined on the Agilent 4200 Tapestation (High sensitivity D1000 kit). Based on the mass of 1 genome of the TB reference strain H37Rv (4,411,532 bp = 5 fg), we estimated the number of genome equivalents per µL (GE/µL) in the quantitated purified fragmented DNA. 1 GE is defined as the mass of fragmented Mtb DNA equal to 5 fg. Dilutions of fragmented DNA were prepared in nuclease-free water and stored at −20 ˚C in lo-bind plasticware.

Non-tuberculosis mycobacteria were cultured using the same methods as Mycobacterium *tuberculosis*. Extractions were carried out using the DNeasy Blood and Tissue Nucleic Acid Extraction Kit using the gram-negative bacteria sample preparation, “Purification of Total DNA from Animal Tissues (SpinColumn Protocol)”. For other bacteria, liquid cultures were grown overnight and extracted using the DNeasy kit as described above.

### cfDNA preparation from clinical samples

The cfDNA from clinical samples (CFM-SA1-11, CFM-UP1-9, SUS 1-6, and HC 1-6) were collected from study participants in South Africa, Uganda or at Palo Alto, CA. Approval was obtained for the collection of samples CFM-UP1-9 and SUS 1-6 from the institutional review board (IRB) at the Uganda National Council for Science and Technology; and for samples CFM-SA1-11 and HC 1-6, IRB approval was obtained from Stanford University. All participants were > 18 years of age and provided written informed consent^28^. Samples from patients with pulmonary TB were confirmed via sputum-based culture and/or the Xpert MTB/RIF assay (Cepheid, Sunnyvale, CA, USA); as well as a cfDNA-based real time PCR assay, which targeted a single 72 bp region of IS6110, as previously reported^28^. Additional healthy control samples (HC 7-26) were obtained from Research Blood Components, LLC (Watertown, MA).

Blood from all samples were collected and cfDNA extracted per the optimized protocol identified and reported previously^28^. Briefly, blood was collected in K_2_EDTA tubes (Becton, Dickinson, Franklin Lakes, NJ), centrifuged at 500 x g for 10 min at room temperature, and the plasma was transferred to a new tube, stored at −80 °C and shipped to Stanford University. cfDNA was extracted from 4 mL of plasma using the Maxwell RSC system (Promega) and the Maxwell RSC large-volume ccfDNA kit. Samples were eluted in 100 µL, of which 10 µL was used in the cfDNA-based qPCR assay and the experiments reported herein, unless otherwise specified.

### Singleplex and genome-wide (multiplexed) PCR amplification

Singleplex and multiplexed PCR amplification was carried out using the Multiplex PCR Plus kit (Qiagen). For singleplex amplification, the final concentration of the single primer pair was 2 µM, and 0.5 x Q solution in 1x QIAGEN Multiplex PCR Master Mix. For multiplexed amplification, the final concentrations in each reaction were 400 nM per primer pair, with a total primer concentration of 7.2 µM and 0.5 x Q solution in 1x QIAGEN Multiplex PCR Master Mix. For general experiments, 1/50 of the volume was DNA template. When using clinical cfDNA isolates, 1/5 of the volume was DNA template in the final reaction. Reactions were incubated at 95 °C for 5 min, followed by 40 cycles of 95 °C for 30 s, 60 °C for 90 s, and 72 °C for 30 s. And finally, 68 °C for 10 min and then held at 4 °C.

### Recombinase Polymerase Amplification (RPA)

RPA Reactions were performed using TwistAmp Basic kits (TwistDx UK). 50 µL reactions were performed as directed by the manufacturer’s protocol. To make other reaction volumes, single-use pellets were rehydrated and pooled to generate a master reaction mix that was distributed into 10–20 µL individual reactions. Final primer concentration was 500 nM of each primer (primer sequences are listed in Table S1). Magnesium acetate (280 mM) was added to the wall of the tube, so that reactions began simultaneously upon centrifugation at 3200 x g. RPA reactions were incubated at 37 °C in a thermocycler for 20 min, with 10 min at 75 °C to inactivate polymerase unless otherwise specified.

### Cas13 detection

*Leptotrichia wadei* Cas13a enzyme was purchased from a commercial vendor (Genscript) and aliquoted into 3 µL aliquots and flash frozen with liquid nitrogen and stored at −80 °C until use. Each crRNA was either synthesized using in vitro transcription or purchased from Synthego Corporation (IS6110_2). LwCas13 was kept on ice until rehydrating in 49 µL of Cas 13 storage buffer (50 mM Tris-HCl at pH 7.5, 600 mM NaCl, and 5% glycerol). The Cas13 detection mix contains Cas Cleavage Buffer (40 mM Tris, 6 mM MgCl2, 1 mM DTT), RNase inhibitor to 1 U/µL (New England BioLabs), T7 polymerase to 1.5 U/µL (New England BioLabs), rNTPs to 1 mM (New England BioLabs), MgCl2 to 9 mM, rehydrated Cas 13 protein to 45 nM, RNase Alert v2 reporter to 125 nM (Life Technologies). The final crRNA concentration in detection reactions was 22.5 nM (1.25 nM of each of 18 crRNAs for pooled detection; 22.5 nM of a single crRNA for parallel detection). For the clinical samples that were detected by lateral flow, the concentration of each crRNA was 12.5 nM, resulting in a total crRNA concentration of 225 nM.

Fluorescent-based Cas13 detection was measured on a Spectramax M5 Plate Reader (Molecular Devices), using 490 nM for the excitation wavelength and 520 nM for the emission wavelength unless otherwise specified. RPA reactions were added to the Cas13 detection mix at a ratio of 1:19; then incubated for 2 h at 37 °C unless otherwise specified.

### Droplet experiment protocol

Droplets experiments were performed as previously described^20^ using the DropArray platform. Briefly, detection sets were prepared at 2.2X final concentration of 45 nM purified *Leptotrichia wadei* Cas13a, 22.5 nM total crRNA concentration (1.25 nM of each of 18 crRNA for pooled detection; 22.5 nM of one crRNA for individual detection), 500 nM quenched fluorescent RNA reporter (RNAse Alert v2, Thermo Scientific), 2 μl murine RNase inhibitor (New England Biolabs) in nuclease assay buffer (40 mM Tris-HCl, 60 mM NaCl, pH 7.3) with 1 mM NTPs and 0.6 μl T7 polymerase mix (New England Biolabs). Amplified samples were diluted 1:10 into nuclease-free water supplemented with 13.2 mM MgCl_2_ prior to barcoding with fluorescent dyes. 20 µL of each sample and detection mix were then emulsified into droplets using a BioRad QX200 droplet generator using fluorous oil (3 M 7500, 70 µL) containing 2% 008-fluorosurfactant (RAN Biotechnologies.) Droplets were pooled and loaded into a DropArray chip, imaged for content identification by fluorescent barcode identification, droplet pairs merged and then incubated at 37 ˚C, and imaged for assay signal at 0, 1 h, and 3 h time points relative to the start of the incubation.

### Lateral flow assay

For lateral flow detection, we used the commercially available lateral flow assay kit, Milenia Genline HybriDetect™ kit (TwistDx) (Fig. S6). In the Cas13 detection step, the RNase Alert v2 fluorescent reporter was replaced with a FAM-Biotin labeled poly-U reporter (5’-FAM-UUUUUUUUUUUUUU-Biotin-3’) (Integrated DNA Technologies) in the Cas13 detection mix to a final concentration of 1 µM; then added to the Hybridetect™ assay buffer at a ratio of 1:4. Lateral flow strips were then inserted into the microtube containing the buffer and incubated for 10 minutes, after which results were quantified using ImageJ software. Normalized Signal was calculated as (Test Band Intensity)/(Test Band Intensity + Control Band Intensity).

### Statistics & reproducibility

Data are shown as original values or median with error bars depicting range and standard deviation. Technical and biological replicates of samples were tested for reproducibility (up to six replicates) and the variability was quantified and is discussed in the manuscript text. For clinical testing, sample size calculations were not performed as comprehensive clinical evaluation is not the goal of this study. Sample size of the experimental group (active and suspected TB) was based on availability of clinical samples. Sample size of the control group (healthy individuals) was chosen to be larger than the experimental group. Experiments were not randomized but the investigators were blinded to allocation during experiments and data analysis. No data were excluded from the analyses.

### Reporting summary

Further information on research design is available in the Nature Portfolio Reporting Summary linked to this article.

## Supplementary information

Supplementary Information

Supplementary Data

Reporting Summary

## Data Availability

The data generated in this study are provided in the Supplementary Information, Source Data, and Supplemental Data files. Sequences information used in our work were all acquired from the publicly accessible NCBI database. All protocols have been described in the Methods section or in references therein. Custom algorithms were used for multiplex primer design and the code is available on Github at https://github.com/gowthamthakku/watson. Source data are provided with this paper.

## Source data

Source Data
