# Supplementary material for: Genome-wide tiled detection of circulating Mycobacterium tuberculosis cell-free DNA using Cas13
Source: Nat Commun. 2023 Mar 31;14:1803. doi: 10.1038/s41467-023-37183-8 (PMC10064635; doi:10.1038/s41467-023-37183-8)
Supplement: Supplementary file 1 — Supplementary Information [file 41467_2023_37183_MOESM1_ESM.pdf]

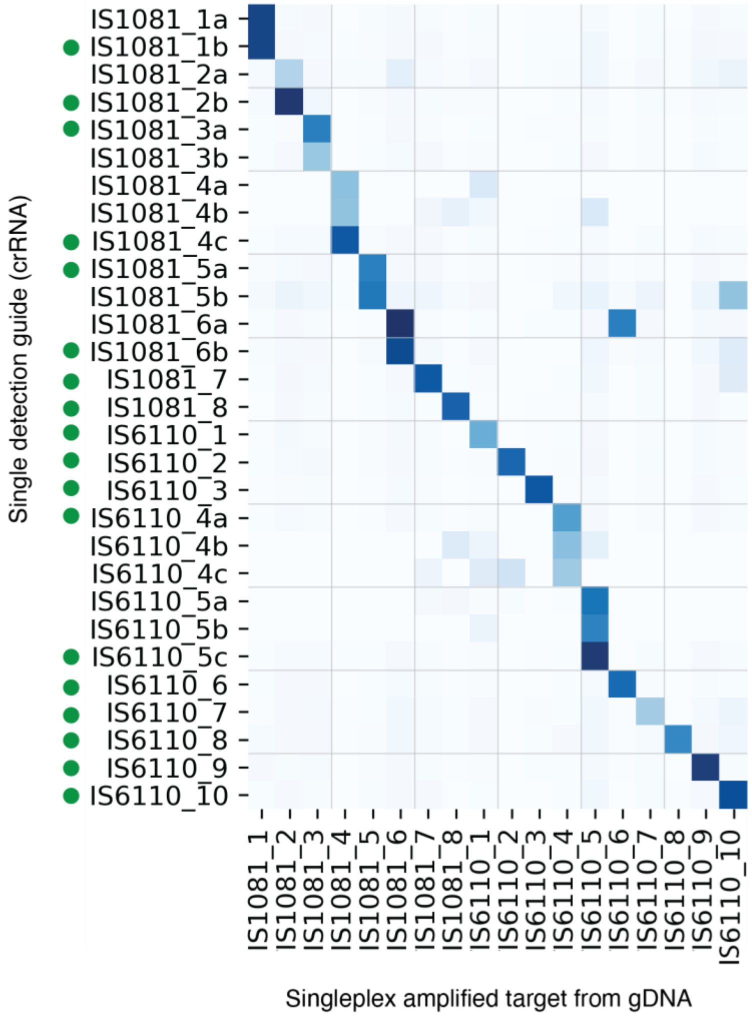

Supplementary Figure S1: Heatmap depicting performance of guides against their corresponding targets; 1-3 crRNA guides were designed for each target, depending on the sequence space between primers; all guides were tested against singleplex amplified targets and the best performing ones were chosen (guides chosen for the final assay are indicated with a green dot and also shown in Figure 1c).

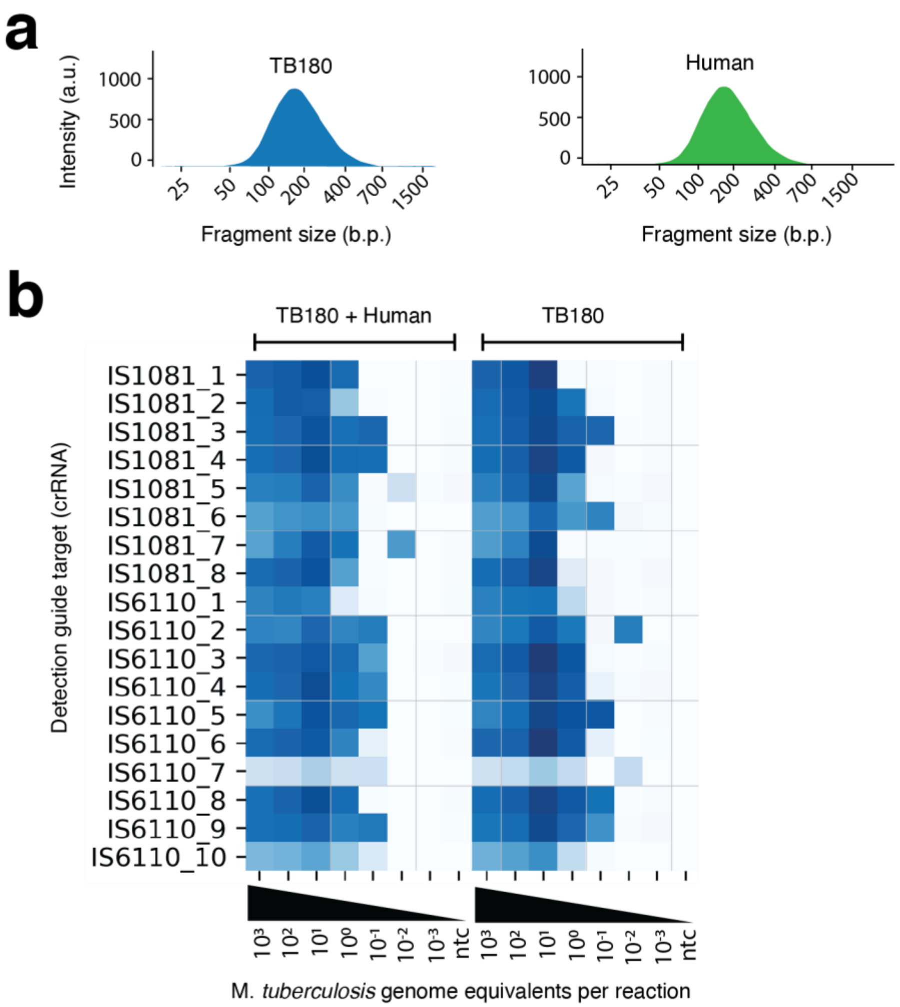

Supplementary Figure S2: a. Size profiles show the representation of fragment size distribution after NEBNext Fragmentase reaction. Fragmentation profiles are shown for *M. tuberculosis* H37Rv genomic DNA and fragmented human genomic DNA. **b.** Heatmap of WATSON using droplet platform over a range of *M. tuberculosis* genomic DNA concentrations for two different materials. The first is fragmented *M. tuberculosis* genomic DNA with an median fragment size of 180bp (shown in **a.** TB180) with a constant background of 1 genomic equivalent per uL of human fragmented gDNA (shown in **a.** Human). The second is the same *M. tuberculosis* genomic DNA without human background.

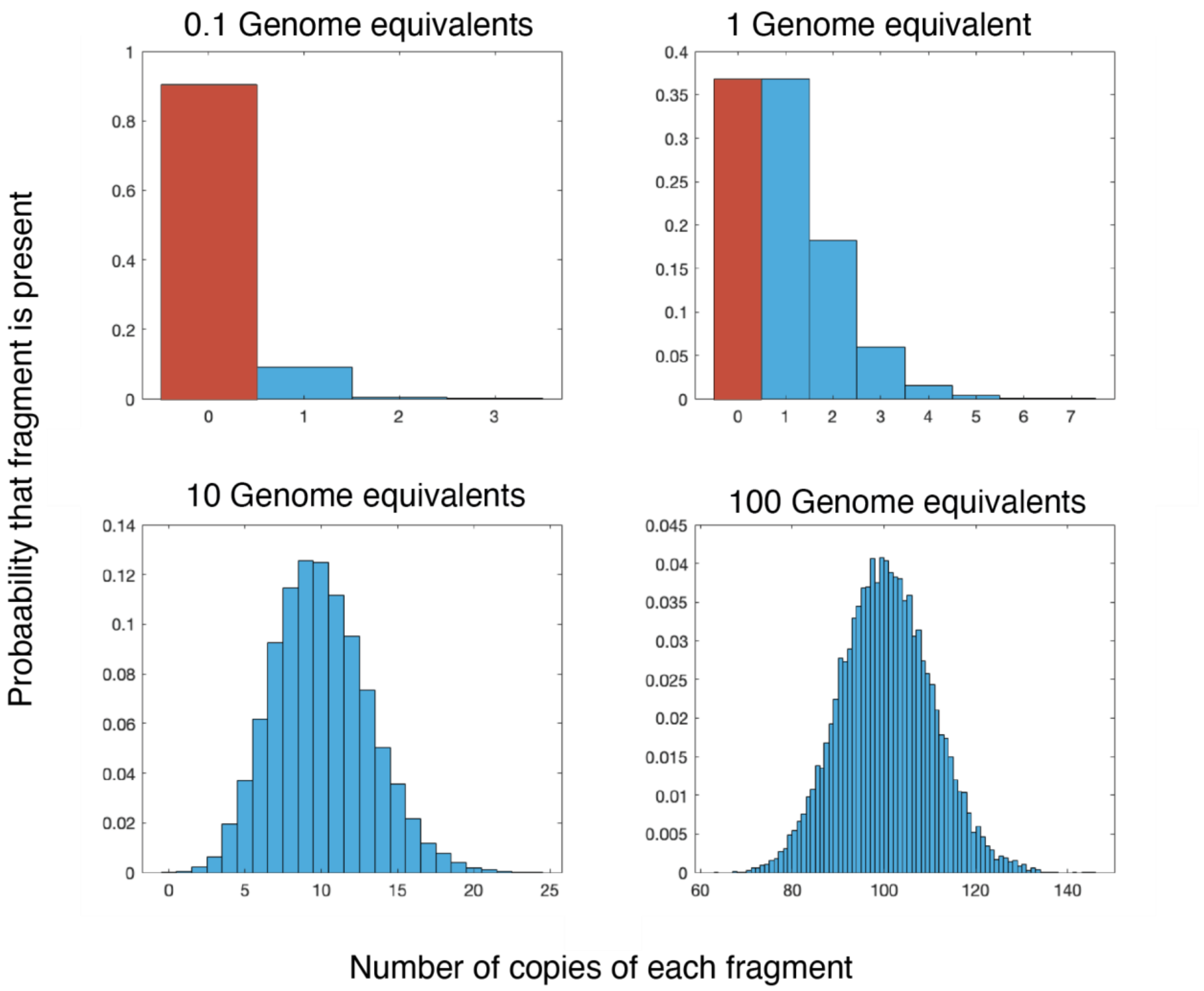

Supplementary Figure S3: Computer simulation of stochastic distribution of cfDNA fragments (assumed to have constant length of 200 bp) when different genome equivalents are considered (genome length assumed to be 4 million bp); at 1 GE (4 million / 200 = 20,000 total fragments), we expect ~35% of fragments to be absent, ~35% of fragments to be present as 1 copy, ~18% of fragments to be present as 2 copies, and so on.

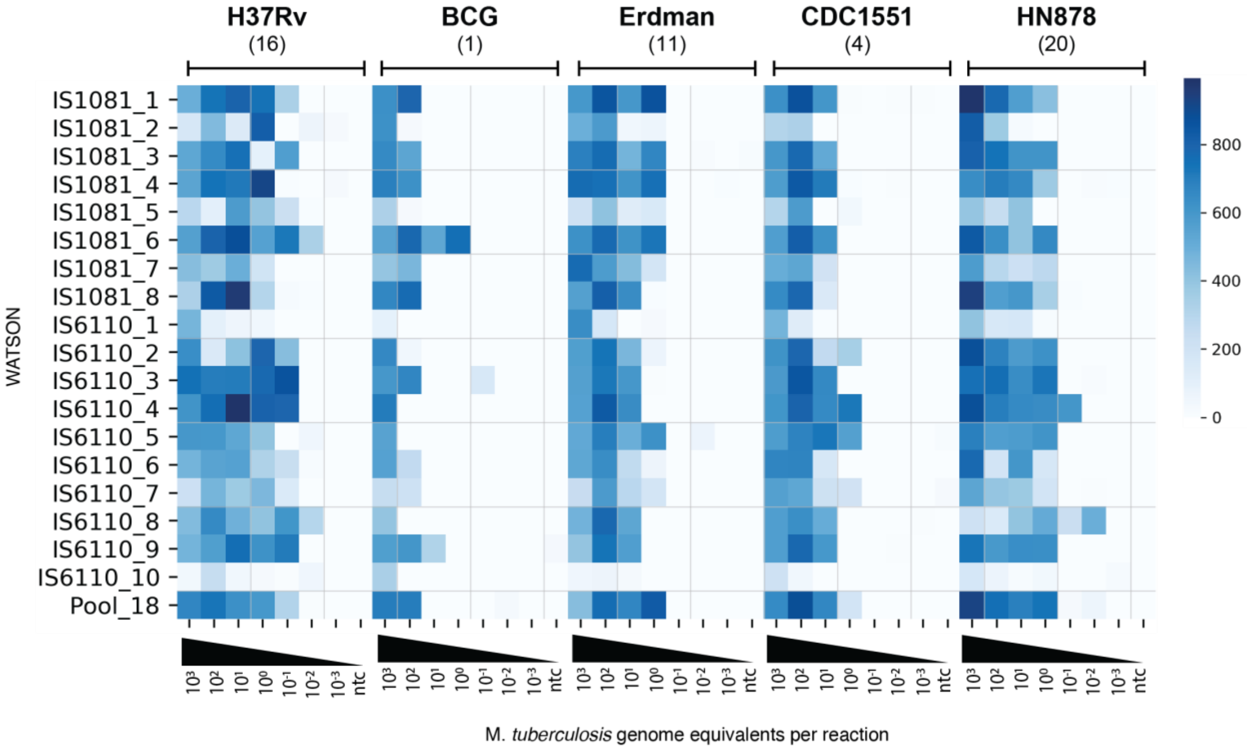

Supplemental Figure S4: Limit of detection of WATSON on 4 strains of *M. tuberculosis* (H37Rv, Erdman, CDC 1551, and HN878, as well as *M. bovis* Bacille Calmette-Guérin (BCG)). The number in parenthesis below each strain indicates the number of copies of IS6110 in that strain. All tests used fragmented gDNA, with an average fragment size of 120-150bp. (ntc = no template control)

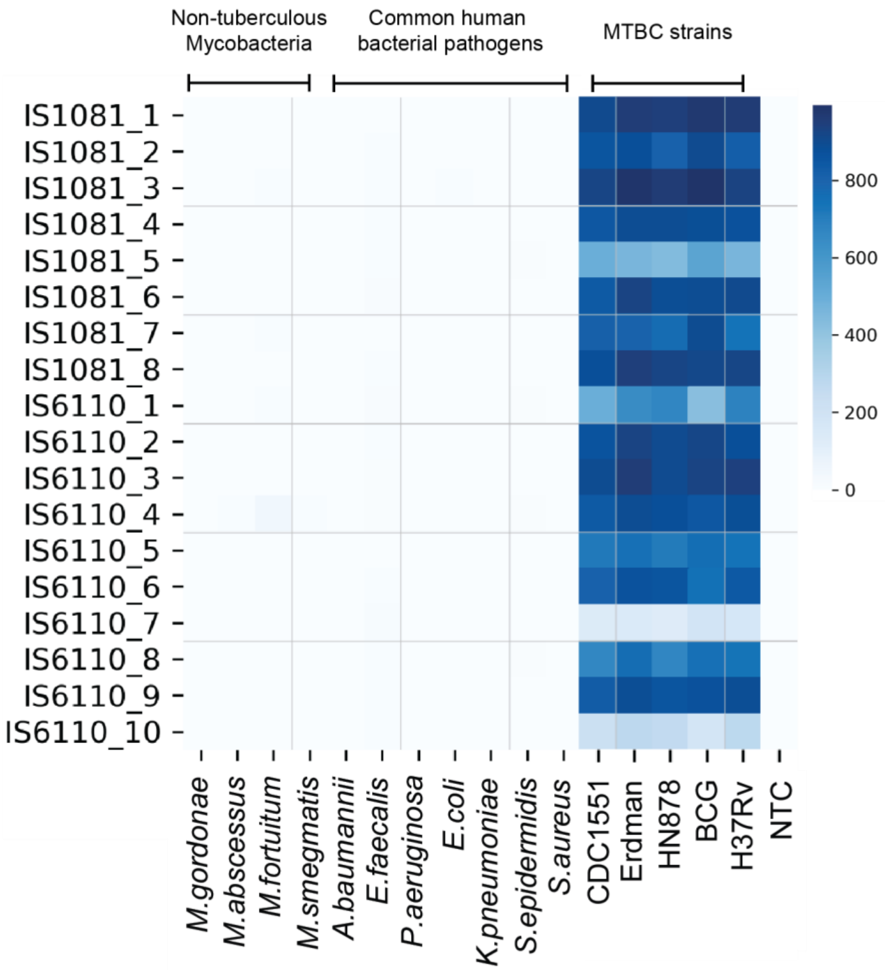

Supplemental Figure S5: Specificity of WATSON as tested against several non-tuberculous mycobacteria and other common human bacterial pathogens; and for strains of the *Mycobacterium tuberculosis* complex (MTBC). 1e4 genome equivalents per reaction was used for each pathogen shown. (NTC = no template control)

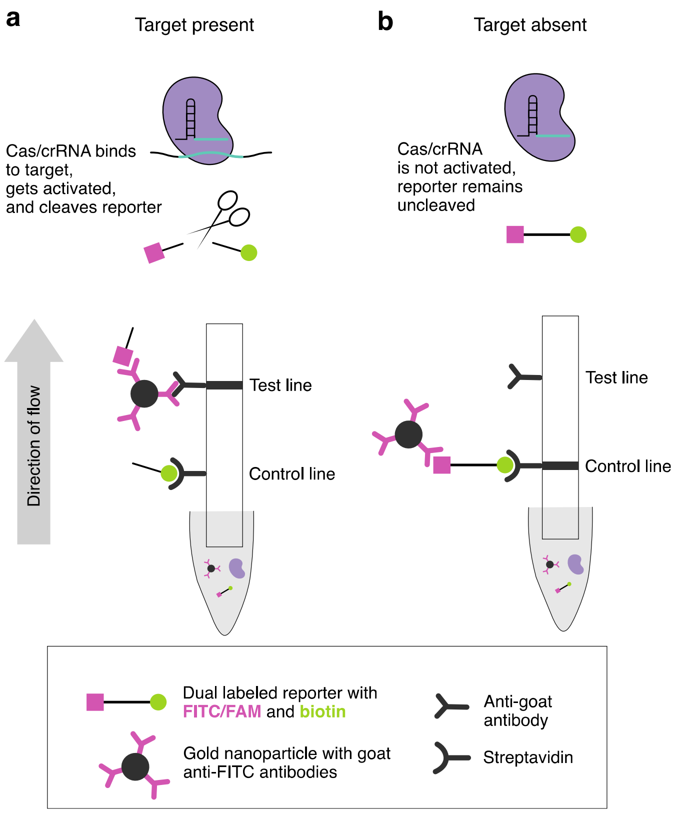

Supplemental Figure S6: Schematic illustration of detecting Cas13 activity on a lateral flow strip. **a**. When nucleic acid target is recognized by the crRNA/Cas13 complex, Cas13 activity is detected as a visible Test line on the lateral flow strip with a weak or no line visible at the Control line. **b.** When nucleic acid target is absent and Cas13 is not activated, only a visible line appears at the Control line.

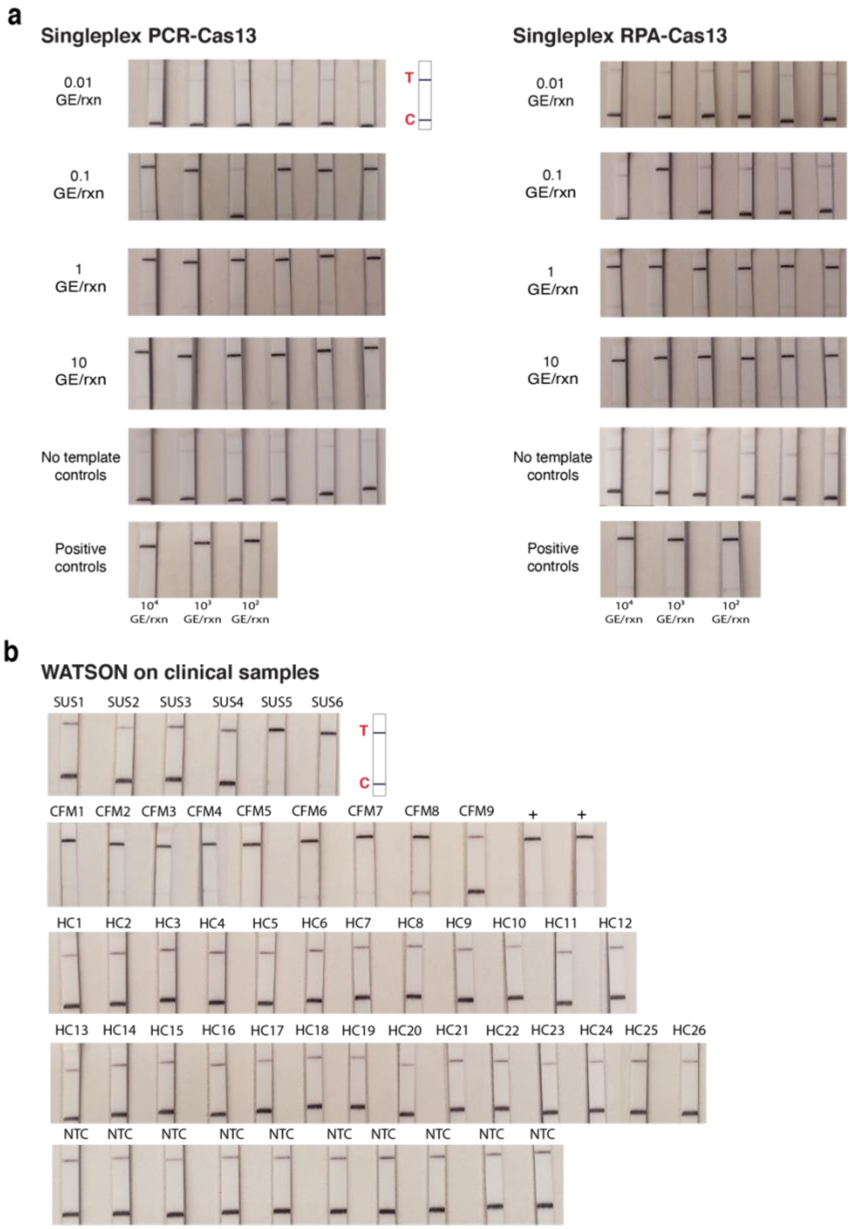

Supplemental Figure S7: Images of lateral flow strips for **a.** a dilution series of fragmented Mtb gDNA that were amplified via singleplex PCR (left) and RPA (right) using a primer pair targeting IS6110_2 combined with CRISPR-Cas13 detection. Both methods of amplification, detection and lateral flow readout show a similar limit of detection of 0.1 – 1 genome equivalents per reaction, which is concordant with the limit of detection using fluorescence readout, as shown in Figure 4a. Six replicates were run at each gDNA concentration; **b.** clinical samples that were amplified and detected by WATSON with a lateral flow readout. cfDNA was extracted from the equivalent of 400 µL of patient plasma. (A schematic of the lateral flow strip is shown where 'T' is the test line and 'C' is the control line, HC = Healthy control, SUS = Suspected TB patient who presented with a cough for more than a week but tested negative for culture and GeneXpert, CFM = Confirmed TB patient who tested positive by culture and/or GeneXpert, NTC = no template control, + = fragmented DNA from H37Rv at 1e2 genome equivalents per reaction.)

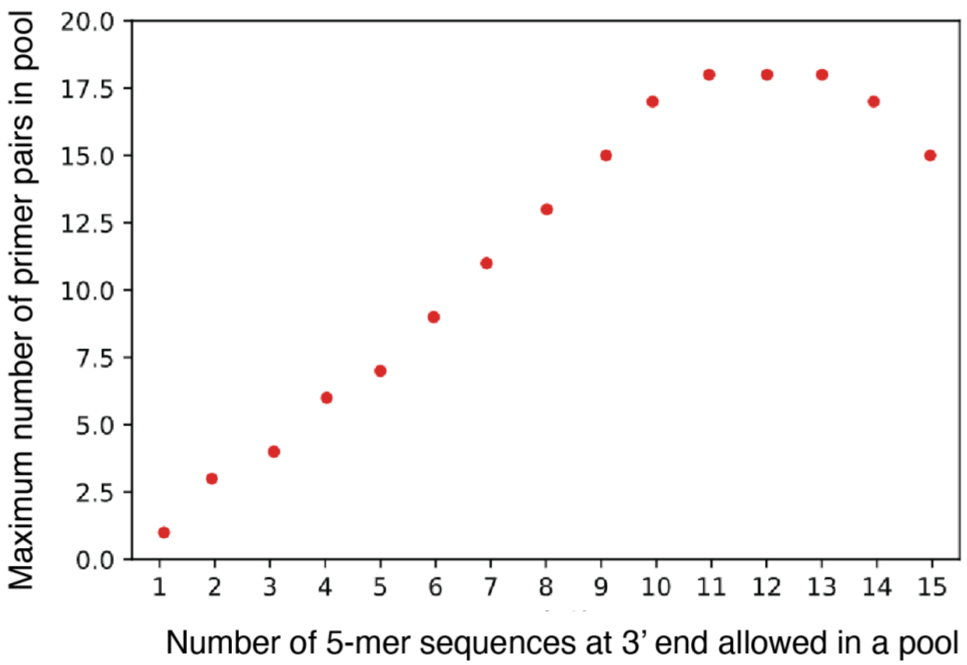

Supplemental Figure S8. Plot of the maximum number of primers in a pool as a function of the number of 5-mer sequences at the 3' end that are allowed in the pool as determined by the primer design rules (See Methods). Primers were designed to target the IS6110 and IS1081 regions and minimize 3'-3' interactions between pairs. The top 100 ranked and filtered 5-mers (as described in the Methods section) were sampled at each number of allowed 5-mer sequences. The maximum number of primers that can be pooled together according to our design rules is 18, where each primer pair has one of 11 5-mer sequences at its 3' end.

Table S1. Primer and guide sequences for WATSON, singleplex SHERLOCK, and singleplex RPA*

Name	Forward Primer Nucleotide Sequence (Underlined Nucleotides are T7 promoter)	Reverse Primer Nucleotide Sequence	Guide RNA template ssDNA Nucleotide Sequence	Description
IS1081-1	GAA ATT AAT ACG ACT CAC TAT AGG GCA AGT CGC AAG TGT CGA TCA TGG CCA AAG A	GGC GAG GAA GGT ATA CGG GCC G	GCTCGACGAAGCCGTAGAGGCGTTTCGGGTTTTAGTCCCCTTCGTTTTTGGGGTAGTCTAAATCCCCTATAGTGAGTCGTATTAATTTC	WATSON Primer Pair (In 18x)
IS1081-2	GAA ATT AAT ACG ACT CAC TAT AGG GAT CAG TTG TTG CCC AAT ATG ATC GGG TAC T	GTC GGT GCG GGC GGT GT	AAACTCCCCGCGGTGGCCGAGCACCTCGGTTTTAGTCCCCTTCGTTTTTGGGGTAGTCTAAATCCCCTATAGTGAGTCGTATTAATTTC	WATSON Primer Pair (In 18x)
IS1081-3	GAA ATT AAT ACG ACT CAC TAT AGG GCT CTT CTC ATC TTA TCG ACG CCG AGC AGC	CGA GAG CAG CCC GCG CAG C	TTCTGGCTGACCAACTCGCACAGGCGAGGTTTTAGTCCCCTTCGTTTTTGGGGTAGTCTAAATCCCCTATAGTGAGTCGTATTAATTTC	WATSON Primer Pair (In 18x)
IS1081-4	GAA ATT AAT ACG ACT CAC TAT AGG GCG GGC TAC CGC GAA CGC AGC	TCG ATG GTT GCG GCA CGG GTG T	AGCGCAACGGCTACCGCCACCGTGATTTGTTTTAGTCCCCTTCGTTTTTGGGGTAGTCTAAATCCCCTATAGTGAGTCGTATTAATTTC	WATSON Primer Pair (In 18x)
IS1081-5	GAA ATT AAT ACG ACT CAC TAT AGG GAA CCC ACT ACG CAG CCA ATC TGA TGG CAG C	TCG GGC TGG TCG TAG ATG GAG TGC AGC	CACCCCGAAGCCCTCCTGGCCGTGGGTGGTTTTAGTCCCCTTCGTTTTTGGGGTAGTCTAAATCCCCTATAGTGAGTCGTATTAATTTC	WATSON Primer Pair (In 18x)
IS1081-6	GAA ATT AAT ACG ACT CAC TAT AGG GCT CAC CCG AGC CCG AGC AGC	CAG TCT AGG TGG TCA GTG CTG GGG TGT	ACTGACCAGCACCGAAGAACCCGCCAAGGTTTTAGTCCCCTTCGTTTTTGGGGTAGTCTAAATCCCCTATAGTGAGTCGTATTAATTTC	WATSON Primer Pair (In 18x)
IS1081-7	GAA ATT AAT ACG ACT CAC TAT AGG GCC GCC GAG GAC GGG GCC G	GCG ACC CCG GAC AGG CCG	CTGGCTGGCGTTCTTCCGCGACCTGGTCGTTTTAGTCCCCTTCGTTTTTGGGGTAGTCTAAATCCCCTATAGTGAGTCGTATTAATTTC	WATSON Primer Pair (In 18x)
IS1081-8	GAA ATT AAT ACG ACT CAC TAT AGG GCC GAC GCC CTG GTG CTC A	ACG CCG GTG GCG ATC AAG GTG T	GGTGCGCGAGGCAGGCCGCGTCGTCGGGGTTTTAGTCCCCTTCGTTTTTGGGGTAGTCTAAATCCCCTATAGTGAGTCGTATTAATTTC	WATSON Primer Pair (In 18x)
IS6110-1	GAA ATT AAT ACG ACT CAC TAT AGG GAA TTG CGA AGG GCG AAC GCG ATT TTA AAG A	ATG AAC CGG GTA ATT AGC GTG CTG GCC G	CCGCGTCGGCTTTCTTCGCGGCCGAGCTGTTTTAGTCCCCTTCGTTTTTGGGGTAGTCTAAATCCCCTATAGTGAGTCGTATTAATTTC	WATSON Primer Pair (In 18x)
IS6110-2	GAA ATT AAT ACG ACT CAC TAT AGG GCC AAC AAG AAG GCG TAC TCG ACC TGA AAG A	CGC TCG CTG AAC CGG ATC GAT GTG TAC T	GTTATCCACCATACGGATAGGGGATCTCGTTTTAGTCCCCTTCGTTTTTGGGGTAGTCTAAATCCCCTATAGTGAGTCGTATTAATTTC	WATSON Primer Pair (in 18x and Singleplex PCR)
IS6110-3	GAA ATT AAT ACG ACT CAC TAT AGG GTC AGC ACG ATT CGG AGT GGG CAG C	CGC ACC GTC TCC GCG CAG C	TCAGTGAGGTCGCCCGTCTACTTGGTGTGTTTTAGTCCCCTTCGTTTTTGGGGTAGTCTAAATCCCCTATAGTGAGTCGTATTAATTTC	WATSON Primer Pair (In 18x)
IS6110-4	GAA ATT AAT ACG ACT CAC TAT AGG GCC GAT GGT TTG CGG TGG GGT GT	CCG GTT GAT GTG GTC GTA GTA GGT CGA TG	CACAGCTGACCGAGCTGGGTGTGCCGATGTTTTAGTCCCCTTCGTTTTTGGGGTAGTCTAAATCCCCTATAGTGAGTCGTATTAATTTC	WATSON Primer Pair (In 18x)
IS6110-5	GAA ATT AAT ACG ACT CAC TAT AGG GTC CAC GCC GCC AAC TAC GGT GT	GAC GGT GCA TCT GGC CAC CTC GAT G	ACGGTGTTTACGGTGCCCGCAAAGTGTGGTTTTAGTCCCCTTCGTTTTTGGGGTAGTCTAAATCCCCTATAGTGAGTCGTATTAATTTC	WATSON Primer Pair (In 18x)
IS6110-6	GAA ATT AAT ACG ACT CAC TAT AGG GGC GCC AGG CGC AGG TCG ATG	CGC CGC AAG CGC TTC AGC TCA	GTCGATGCCGGCGCACGGCCCGGGACCAGTTTTAGTCCCCTTCGTTTTTGGGGTAGTCTAAATCCCCTATAGTGAGTCGTATTAATTTC	WATSON Primer Pair (In 18x)
IS6110-7	GAA ATT AAT ACG ACT CAC TAT AGG GTG GCG GGT CGC TTC CAC GAT G	TCT TGT TGG CGG GTC CAG ATG GCT T	CCACCTCCATGGTCCTCGACGCGATCGAGTTTTAGTCCCCTTCGTTTTTGGGGTAGTCTAAATCCCCTATAGTGAGTCGTATTAATTTC	WATSON Primer Pair (In 18x)
IS6110-8	GAA ATT AAT ACG ACT CAC TAT AGG GCG GTC GGA GCG GTC GGA AGC TC	TTG ATC AGC TCG GTC TTG TAT AGG CCG	TATGACAATGCACTAGCCGAGACGATCAGTTTTAGTCCCCTTCGTTTTTGGGGTAGTCTAAATCCCCTATAGTGAGTCGTATTAATTTC	WATSON Primer Pair (In 18x)
IS6110-9	GAA ATT AAT ACG ACT CAC TAT AGG GAT GCA CCG TCG AAC GGC TGA TGA CCA A	AGC GAT CGT GGT CCT GCG GGC TT	CTCGGCCTGTCCGGGACCACCCGCGGCAGTTTTAGTCCCCTTCGTTTTTGGGGTAGTCTAAATCCCCTATAGTGAGTCGTATTAATTTC	WATSON Primer Pair (In 18x)
IS6110-10	GAA ATT AAT ACG ACT CAC TAT AGG GTG GAA AGG ATG GGG TCA TGT CAG GTG GT	CAT CCG CAC CGC CCG CTC A	TCATCGAGGAGGTACCCGCCGGAGCTGCGTTTTAGTCCCCTTCGTTTTTGGGGTAGTCTAAATCCCCTATAGTGAGTCGTATTAATTTC	WATSON Primer Pair (In 18x)
RPA-IS6110-2	GAA ATT AAT ACG ACT CAC TAT AGG GAA CAA GAA GGC GTA CTC GAC CTG AAA GA	GCT CGC TGA ACC GGA TCG ATG TGT ACT	GTTATCCACCATACGGATAGGGGATCTCGTTTTAGTCCCCTTCGTTTTTGGGGTAGTCTAAATCCCCTATAGTGAGTCGTATTAATTTC	RPA Singleplex Primer Pair

* Underlined nucleotides represent the T7 promoter sequence necessary for Cas13 Detection
