# Supplementary material for: Genome-wide tiled detection of circulating Mycobacterium tuberculosis cell-free DNA using Cas13
Source: Nat Commun. 2023 Mar 31;14:1803. doi: 10.1038/s41467-023-37183-8 (PMC10064635; doi:10.1038/s41467-023-37183-8)
Supplement: Supplementary file 3 — Reporting Summary [file 41467_2023_37183_MOESM3_ESM.pdf]

nature portfolio

Corresponding author(s): Deborah T. Hung

Last updated by author(s): Feb 14, 2023

Reporting Summary

Nature Portfolio wishes to improve the reproducibility of the work that we publish. This form provides structure for consistency and transparency in reporting. For further information on Nature Portfolio policies, see our Editorial Policies and the Editorial Policy Checklist.

Statistics

For all statistical analyses, confirm that the following items are present in the figure legend, table legend, main text, or Methods section.

n/a	Confirmed	
☐	☒	The exact sample size (*n*) for each experimental group/condition, given as a discrete number and unit of measurement
☐	☒	A statement on whether measurements were taken from distinct samples or whether the same sample was measured repeatedly
☒	☐	The statistical test(s) used AND whether they are one- or two-sided<br>*Only common tests should be described solely by name; describe more complex techniques in the Methods section.*
☒	☐	A description of all covariates tested
☐	☒	A description of any assumptions or corrections, such as tests of normality and adjustment for multiple comparisons
☐	☒	A full description of the statistical parameters including central tendency (e.g. means) or other basic estimates (e.g. regression coefficient) AND variation (e.g. standard deviation) or associated estimates of uncertainty (e.g. confidence intervals)
☒	☐	For null hypothesis testing, the test statistic (e.g. *F*, *t*, *r*) with confidence intervals, effect sizes, degrees of freedom and *P* value noted<br>*Give P values as exact values whenever suitable.*
☒	☐	For Bayesian analysis, information on the choice of priors and Markov chain Monte Carlo settings
☒	☐	For hierarchical and complex designs, identification of the appropriate level for tests and full reporting of outcomes
☐	☒	Estimates of effect sizes (e.g. Cohen's *d*, Pearson's *r*), indicating how they were calculated

Our web collection on statistics for biologists contains articles on many of the points above.

Software and code

Policy information about availability of computer code

Data collection	No software was used for data collection.
Data analysis	Data analysis included the following open-source software, Bowtie2. Custom algorithms were used for multiplex primer design and the code is available on Github at https://github.com/gowthamthakku/watson

For manuscripts utilizing custom algorithms or software that are central to the research but not yet described in published literature, software must be made available to editors and reviewers. We strongly encourage code deposition in a community repository (e.g. GitHub). See the Nature Portfolio guidelines for submitting code & software for further information.

Data

Policy information about availability of data

All manuscripts must include a data availability statement. This statement should provide the following information, where applicable:

- Accession codes, unique identifiers, or web links for publicly available datasets
- A description of any restrictions on data availability
- For clinical datasets or third party data, please ensure that the statement adheres to our policy

The data generated in this study are provided in the Supplementary Information, Source Data, and Supplemental Data files. Sequences information used in our work were all acquired from the publicly accessible NCBI database. All protocols have been described in the Methods section or in references therein. Custom algorithms were used for multiplex primer design and the code is available on Github at https://github.com/gowthamthakku/watson

Human research participants

Policy information about studies involving human research participants and Sex and Gender in Research.

Reporting on sex and gender	This information has not been collected.
Population characteristics	The population characteristics used in this study include disease status (confirmed TB, suspected TB, no TB) and the results from confirmatory microbiology tests. All participants were >18 years of age. Other covariates (eg. sex and gender) were not collected.
Recruitment	Participants from the Ugandan cohort, who were diagnosed with TB were consecutively recruited for the study as long as their plasma volume was sufficient for the study. We did not have any other selection criteria and therefore unlikely to have introduced any selection bias. Samples from participants from the South African cohort were collected consecutively from adults meeting WHO presumptive TB criteria who provided consent. There is unlikely to be any bias as these types of patients are commonly tested for TB in our setting.
Ethics oversight	Approval was obtained from the institutional review board (IRB) at the Uganda National Council for Science and Technology and from Stanford University.

Note that full information on the approval of the study protocol must also be provided in the manuscript.

Field-specific reporting

Please select the one below that is the best fit for your research. If you are not sure, read the appropriate sections before making your selection.

☒ Life sciences ☐ Behavioural & social sciences ☐ Ecological, evolutionary & environmental sciences

For a reference copy of the document with all sections, see nature.com/documents/nr-reporting-summary-flat.pdf

Life sciences study design

All studies must disclose on these points even when the disclosure is negative.

Sample size	For clinical testing, sample size calculations were not performed as comprehensive clinical evaluation is not the goal of this study. Sample size of the experimental group (active and suspected TB) was based on availability of clinical samples. Sample size of the control group (healthy individuals) was chosen to be larger than the experimental group.
Data exclusions	No data were excluded from the analyses.
Replication	Technical and biological replicates of samples were tested for reproducibility (up to six replicates) and the variability was quantified and is discussed in the manuscript text. Data are shown as original values or median with error bars depicting range and standard deviation.
Randomization	Experiments were not randomized but the investigators were blinded to allocation during experiments and data analysis.
Blinding	Samples were coded during experimental data collection, thereby making them blind to the operator/investigator during data collection and analysis. Upon analysis, codes were matched up to original identifiers.

Reporting for specific materials, systems and methods

We require information from authors about some types of materials, experimental systems and methods used in many studies. Here, indicate whether each material, system or method listed is relevant to your study. If you are not sure if a list item applies to your research, read the appropriate section before selecting a response.

Materials & experimental systems

n/a	Involved in the study	
☒	☐	Antibodies
☒	☐	Eukaryotic cell lines
☒	☐	Palaeontology and archaeology
☒	☐	Animals and other organisms
☒	☐	Clinical data
☒	☐	Dual use research of concern

Methods

n/a	Involved in the study	
☒	☐	ChIP-seq
☒	☐	Flow cytometry
☒	☐	MRI-based neuroimaging
